# The emergence of social gaps in mental health: A longitudinal population study in Sweden, 1900-1959

**DOI:** 10.1371/journal.pone.0232462

**Published:** 2020-04-30

**Authors:** Johan Junkka, Glenn Sandström, Lotta Vikström

**Affiliations:** 1 Centre for Demographic and Ageing Research, Umeå University, Umeå, Sweden; 2 Department of Historical, Philosophical and Religious Studies, Umeå University, Umeå, Sweden; Montana State University, UNITED STATES

## Abstract

During the recent decades, social inequalities in mental health have increased and are now one of the most persistent features of contemporary society. There is limited knowledge about when this pattern emerged or whether it has been a historically fixed feature. The objective of this study was to assess whether socioeconomic and gender gaps in mental health changed during the period 1900–1959 in Sweden. We used historical micro data which report all necessary information on individuals' demographic characteristics, occupational attainment and mental disorders (N = 2,450) in a Swedish population of 193,893. Changes over time was tested using multilevel Cox proportional hazard models. We tested how gender-specific risks of mental disorder changed and how gender-specific socioeconomic status was related to risks of mental disorder later in life. We found a reversal in gender gaps in mental health during the study period. Women had a lower risk than men in 1900 and higher risks in 1959. For men, we found a negative gradient in SES risks in 1900 and a positive gradient in 1959. For women, we found no clear SES gradient in the risk of mental disorder. These findings suggest that the contemporary patterns in socioeconomic and gender gaps in mental disorder emerged during the 1940s and 1950s and have since then persisted.

## Introduction

Across the globe, social inequalities in mental health have increased and are now one of the most persistent features of contemporary society. In the western world, women are reported to have worse mental health than men, and poor people suffer more than rich [1–4]. Yet, the historical origin of these inequalities is largely uncertain, primarily, due to the lack of longitudinal population-based studies on mental disorder risks before the 1950s. This study uses Swedish longitudinal microdata to investigate whether socioeconomic and gender inequalities in mental health are persistent features of health disparities or if these inequalities changed during the period 1900–1959.

Numerous studies demonstrate that women run higher risks of mental disorders than men and that women with low socioeconomic status (SES) are the most exposed [1,2,5,6]. Sweden is no exception; since the 1990s socioeconomic and gendered gaps in self-rated mental health and psychiatric diagnosis have widened [7]. Historical studies based on admission rates in 19th- and early 20th-century Europe show inconsistent results, women are in some studies more numerous [8,9], and in others, have lower admission rates until the 1920s [10,11].

Gender gaps have been attributed both to biological and social factors. Neurobiological studies show that women have larger increases in stress response systems than men, contributing to the higher prevalence of stress-related mental disorders among women [12,13]. Sociological studies find that women are more exposed to situations that induce stress, such as lack of trust, financial insecurity and lack of independence which increases the risk of mental disorders [14–16]. However, gender gaps in the overall burden of distress are relatively small [17]. These gaps have been explained by differences in role-based responses to psychological symptoms [15,18,19], caused by gendered perceptions of accepted behaviours. Women are more inclined to internalise problems and express them in the form of depression or anxiety, while men tend to externalise problems and express them through substance abuse, alcohol abuse and antisocial behaviours [14,18].

The negative relationship between SES and mental health is one of the most replicated findings in social sciences and epidemiology. Globally, the gradient is reported across a variety of SES indicators—education, income, occupation—and many types of mental disorders, such as depression, antisocial or substance use disorders [3,4,20–22]. Social causation theory assumes that low SES groups have poorer mental health due to greater socioeconomic deprivation leading to higher levels of psychosocial stress [23,24]. The alternative explanation is that poor mental health leads to a loss of social status, where people who have mental disorders experience a social downward drift as they have higher risks of economic insecurity, such as unemployment.

The inverse relationship between SES and mental disorders can be traced as far back as the 19th century. In a pioneering work from 1855, Jarvis finds that poverty was associated with lunacy in Massachusetts, suggesting that lunacy causes poverty [25]. Similar associations are found for incarceration rates at 19th-century British asylums [9,26,27]. In the 1930s, Faris and Dunham found an ecological connection in Chicago, according to which people from poor neighbourhoods ran higher risks of hospitalisation than others [28]. The inverse relationship between SES and mental disorders is widely reported in studies of post-1940s populations [29–31]. In addition, SES has been shown to have different levels of effect on different mental disorders depending on gender [6,14]. Given the greater differences in working conditions of men and women historically, it is necessary to separately analyse the relationship between SES risks of mental disorder for men and women.

As the economic deprivation was even worse in historical populations, one can assume that the SES gaps were larger before the emergence of the welfare state. However, greater deprivation has not always been linked to poorer health historically. In contrast to contemporary societies [32], past populations demonstrate a positive relationship between life expectancy and SES among men and the expected negative relationship for women [33–35].

Although previous empirical studies on pre-1950s populations have found an inverse relationship between SES and mental disorders, they have not been able to differentiate between social drift and social causation. This is because these studies rely on prevalences in treated samples rather than longitudinal population-based estimates [36]. Furthermore, few studies take into account gender differences. The lack of longitudinal micro-data containing information on mental disorders has limited the possibilities to conduct historical studies of SES and gender variations in mental disorders. This study leverages a unique source in terms of digitised Swedish historical parish registers, that contains information on gender, occupational careers and mental disorders. Using this longitudinal data, we investigated how gender and SES were related to the risk of a mental disorder from age 15 and onward. Thus, we contribute new knowledge on the long-term development of the association between mental health and gender as well as the role of social causation mechanisms for gender-specific SES inequalities in mental health. The associations were explored using multilevel Cox proportional hazard regression that enabled us to control for other selection mechanisms such as age, marital status and socioeconomic context.

### Aim

The overall aim of this study was to examine whether gender and socioeconomic differences in risks of mental disorders varied over time. Based on previous studies we hypothesised (1) that women ran higher risks of mental disorders than men; (2) that low-status groups ran higher risks than high status groups and (3) that the relative differences in mental disorder risks between groups were constant over time.

## Method

### Research design and data

**Fig 1:**
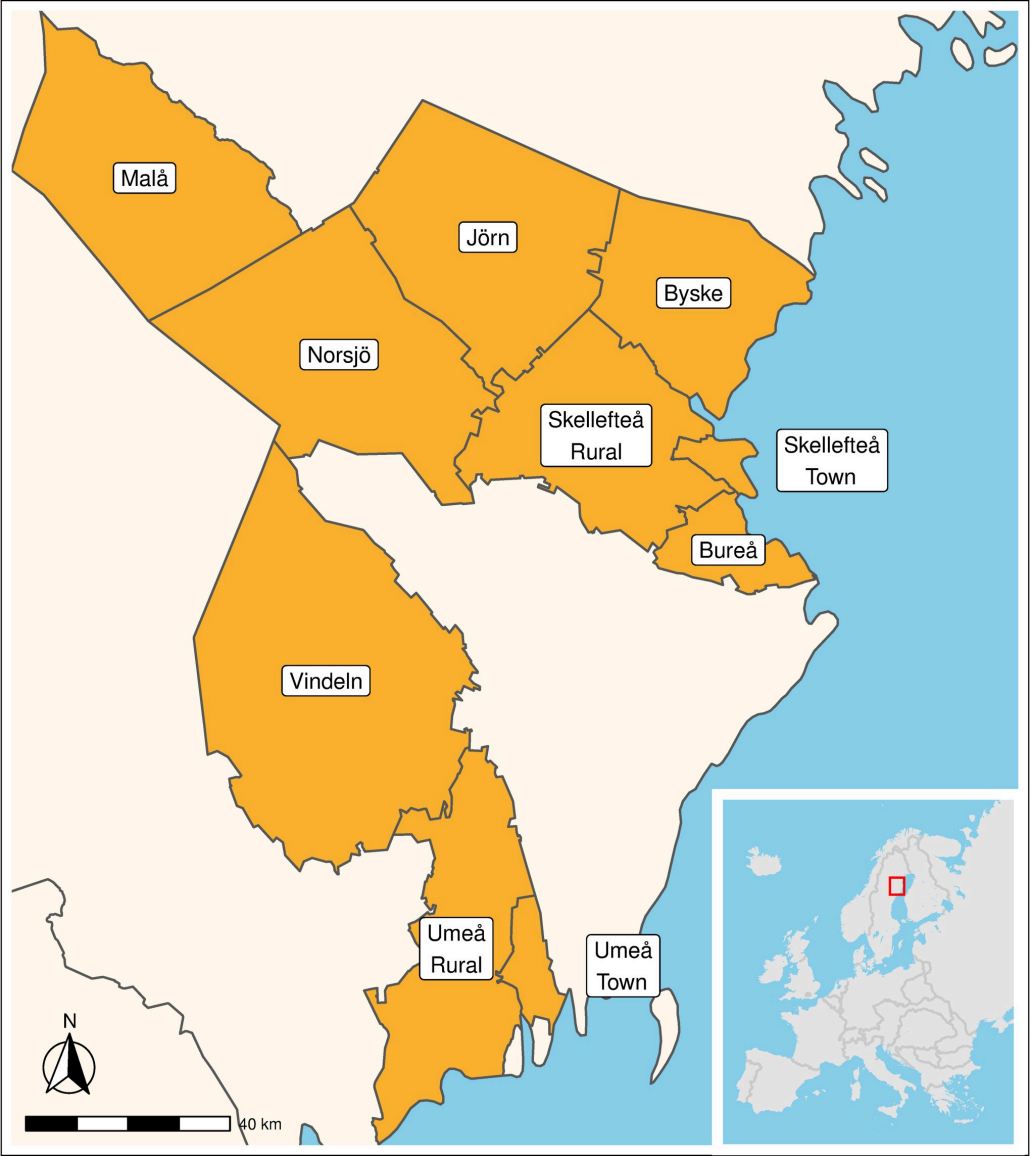
Historical boundaries of parishes within the study area. Source: Based upon administrative boundary data from the Swedish National Archive (Riksarkivet), CC licence.

We applied a longitudinal retrospective design using microdata from the POPLINK database, hosted by the Demographic Data Base (DDB), Umeå University [37]. Ethics approval to conduct this study using the POPLINK data has been obtained from the Regional Ethics Board in Umeå (2015/192-31Ö; 2016/140-32). The data was accessed in a pseudo anonymised format, where direct personal information had been removed, such as names and personal ID number. Thus, informed consent was not needed to conduct the study. The population consisted of all individuals residing in 12 parishes surrounding two small towns, Skellefteå and Umeå, located around the outlets of major rivers at the shores of the Gulf of Bothnia, (Fig 1). The population grew from around 80,000 in 1900 to 160,000 in 1959. During the study period, the region experienced extensive economic and social transformations—the production shifted from being primarily based on agriculture to depend on industrial production [38].

Until the 1950s, population registers were kept by the Swedish Church. Specifically, it was the ministers’ duty to record statistical information on the population in their parish on behalf of Statistics Sweden. For the sample area, these sources have been digitised and linked on an individual level creating a historical longitudinal population register. The database contains continuous information with dates on not only birth, deaths and marriages but also on migration, household structure, occupation and impairment (*Lyte*) [37]. In order to perform these duties, the ministers followed detailed instructions provided by the state on how to record and categorise vital information such as migration, occupational titles and physical or mental health impairments, which made the individuals partly or completely unable to work. These instructions were printed, taught at the minister education and updates were distributed among the ministers throughout the country [39].

The sample consisted of all individuals residing in the region aged 15 to 89 years old, who did not have a mental disorder when entering the study, N = 193,893. Over the life course, 2,450 individuals were reported as having a mental disorder. This was an open cohort where individuals entered the study as they turned 15 years or older, between 1900 and 1949, followed until 1959. Individuals excluded from the sample were a small number of cases that had a mental disorder notation when entering the study. By using the temporal order of events in the longitudinal data, we could ascertain if the reporting of a disorder occurred after attaining a particular SES. The overarching research design was thus to examine how gender and SES experienced prior in life was related to the risk of mental disorder in subsequent years of the life course of men and women.

### Measurement of mental disorder

We defined an individual with a mental disorder as someone who was reported with their first mental disorder in the parish registers between 1900–1959. As these registers retain detailed information on historical population health, they have been extensively used by scholars to study diseases such as smallpox, polio and venereal disease [40–46]. Comparisons of the parish registers with other sources have found that, although officials' fail to define the exact causes of unhealthy conditions, the symptoms reported are sufficient to identify health status by contemporary standards [43,47–51]. In the parish registers, the ministers recorded impairments of all parishioners—information which has been thoroughly examined by disability scholars [52–55]. It should be noted that the most common reason for recording a mental disorder was that the individual was confined to a psychiatric institution. Thus, the record of an individual being mentally ill was often the result of being diagnosed accordingly by a medical professional. Governed by the instructions, the ministers should differentiate between people who were blind, deaf, mute, deafmute, epileptic, physically disabled, intellectually disabled and mentally ill.

Regarding the two latter impairments, the instructions obliged the minister to distinguish between mental illness (*sinnessjukdom*) and feeblemindedness (*sinnesslöhet*). The instructions define someone with a mental illness as having lost their mind, while feebledminded persons were regarded as being mentally ill since childhood [39]. It is clear from the instructions that the minister should separate between congenital disorders (intellectual disabilities) and those that were attained through life (mental disorders, such as depression, antisocial disorders or schizophrenia). In the sources, mental illness was portrayed as any condition where the emotional, cognitive or behavioural state of an individual was disrupting their ability to fulfil their social roles, especially to work and support themselves. Interestingly, this historical perception resembles contemporary definitions of mental disorders, such as the definition used by APA [56].

For the most part, the parish office used this typology to classify individuals when information on mental disorders was recorded. However, ministers also used older derogatory terms to describe people with mental disorders such as insane (*galen*), and increasingly over time, a discriminating terminology such as melancholic, neurotic, psychopath, psychosis, suicidal and schizophrenia. We used keyword searches to classify the disability notations into seven groups: blind, deaf, mute, epileptic, physical disability, intellectual disability and mental disorder. The keyword classification was tested and verified using unsupervised topic modelling, a Latent Dirichlet Allocation (LDA) model [57,58]. The LDA model confirms the groups created by the keyword classification, showing that the classification reflects the patterns of co-occurring words in the notes, which enabled us to distinguish mental disorders from other disability notations.

**Fig 2:**
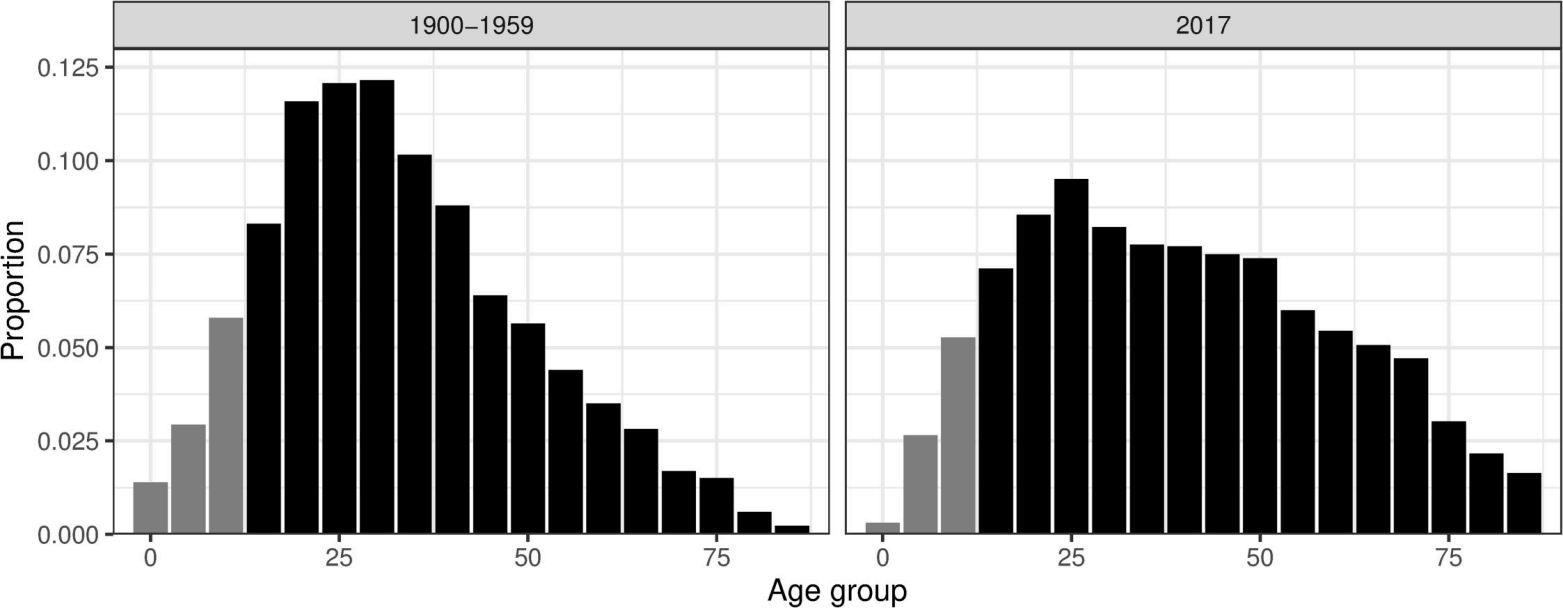
Age distribution of first reported mental disorder in Västerbotten, Sweden, 1900–1959, compared to Sweden in 2017. Sample population coloured in black.

In this study, we were interested in the first occurrence of a mental disorder notation, which primarily occurred after age 15, with a peak between ages 20 and 35 (Fig 2). We compared the age distribution in the historical sample to the modern one using data from the Global Burden of Disease study [59]. As seen in Fig 2, the age distribution of first reported mental disorder in Sweden in 2017 peaked around age 25, similarly to the historical sample. However, in the modern data, the incidence in the older age groups was much higher. This is likely in part produced by increased longevity among individuals with mental illness. We know that mortality was very high among individuals confined to asylums in Sweden during the first half of the 20th-century [60,61].

Additionally, suicide rates have fallen substantially in Sweden, especially since the 1980s [62]. Also, Sweden has a rapidly ageing population and some of the periodic differences in Fig 2 are likely caused by changes in population structure. The distribution of age at first reported mental disorder was consistent with the median onset age of mood disorder, anxiety disorders and substance abuse, but later than the onset of behavioural disorders such as ADHD which occur prior to age 15 [63]. Although performed in different settings, the church officials’ reporting of mental disorder in the first half of the 20th century mirrored that of modern-day register data, at least in terms of age of onset. This age pattern is associated with biological mechanisms related to the development of the brain during adolescence [64]. The similarities of the age distributions across time underlines the usefulness of the historical data for the purpose of exploring social differences in the relative risk of mental disorder.

### Measurements of socioeconomic status

SES was derived from occupational information in the parish registers. Occupation is time-varying, changing across the life course. Due to the longitudinal design of the data, it was possible to follow occupational careers of individuals. This enabled us to identify when in life a person had a specific occupation, and most importantly, what occupation they had before being diagnosed with a mental disorder. SES was attained by classifying the occupational titles into HISCO codes, a historical version to the ISCO scheme, and then grouping them into socioeconomic status groups using the Social Power (SOCPO) scheme [65,66]. The SOCPO scheme divides the occupations into six hierarchical groups, from Elite to Unskilled workers. To create larger, and more comparable groups, we combined the six groups into four and added another group of those without any occupational information (see S1 Table).

### Measurements of confounding factors

Although the longitudinal design enabled a temporal separation of SES exposure and first reported mental disorder, the different groups have different social and contextual compositions (see S2 Table for a description of compositional differences in the sample). The estimates are balanced for a number of confounding factors which could induce selection bias. The risk of a mental disorder and achieving higher SES peaked at different ages, thus each risk-set was conditioned on achieving a certain age by using age as the time scale. Migration can also confound the estimates; healthy individuals are often more likely to migrate and have better occupational careers than stayers [67]. Hence, we differentiate between stayers and migrants. Marital status also has a significant impact on mental health. While being married has a positive effect on mental health, permanent singlehood, divorce and widowhood increase the risks of attaining disorders. The reverse association also exists; poor mental health is associated with being unmarried and higher risks of divorce [19]. Importantly, marital rates had significant socioeconomic patterns [68] and could thus be a potential mechanism confounding the SES mental health relationship. Finally, socioeconomic groups are not evenly distributed geographically. People tend to cluster together with people who are similar to themselves [69]. Low SES individuals have a higher likelihood to live and move to a low SES area. Furthermore, low SES areas often have lower levels of trust and social cohesiveness, which in turn increases risks of poor mental health [70]. This selection effect was handled by balancing the estimated risks for contextual factors such as population density on a neighbourhood level as well as parish specific and neighbourhood specific risks.

The sampled area consisted of 12 parishes and the population was dispersed over 1,311 villages and towns. All individuals in the sample had a recorded parish of residence. However, about 7 percent had, at some point in their life, no recorded place of residence; either because of high levels of residential mobility or due to lack of a permanent residence. In the analysis, individuals without a place of residence were treated as their own geographical group. Neighbourhoods were created by subdividing each parish into smaller areas consisting of neighbouring villages and towns. The process, which followed the same methodology developed in previous studies of the region [71], created 229 neighbourhoods, (see S3 Table for further details on the geographical units). Local socioeconomic structures were constructed by calculating the yearly SES distribution on a neighbourhood level, and then classifying each parish by year into one type of distribution using k-means clustering, creating four types of local SES structures: Urban, Semi-urban, Working-class and Farmers. Individuals without a known place of residence besides being present in the parish were categorised as a fifth group.

### Statistical analysis

Before testing the associations between SES and mental disorders using regression-based methods, we explored the differences in prevalence by SES and gender. Yearly prevalence rates were obtained by calculating the proportion of people who had ever been reported with a mental disorder in relation to the total population at risk that year.

We tested how SES and gender were related to mental disorders using multilevel Cox proportional hazard models. Individuals entered the study when they were first observed in the sources from age 15 and followed until they received their first reported mental disorder or until they were censored, at migration out of the sample area, death, end of the study period in 1959 or until age 90. Variables were not measured at baseline but measured as time-varying, capturing changes over the life course as updates were made to the parish register in terms of changes in occupational status and impairment. Each life course was split at each year to capture how SES and confounders changed over the individual life course as well as over calendar time.

We tested for overall gender differences as well as gender-specific socioeconomic differences. Variations over time were tested by interacting gender and SES with calendar time. The models were tested for non-linearity and non-proportionality. We further tested the interactions with calendar-time for non-linearity by specifying calendar time as a nonlinear spline and stratified by decade. Although there was a slight curvilinear effect of calendar-time, the nonlinear specifications did not add any significant explanatory power to the model or change the relative differences between gender and SES groups in comparison to a specification of calendar-time as a linear function. To assess how the differences changed over the study period we extracted the predicted relative hazard for women compared to men, and in the gender-specific socioeconomic analysis compared to the Elite/Middle Class, the highest SES group in the sample. The predicted hazard ratio was calculated for the full span of calendar time from 1900 to 1959 while adjusting for the mean values of confounders.

The multilevel models allowed us to make these estimates while controlling for both confounding individual differences as well as contextual differences at different geographical levels. The effect of migration, marital status, neighbourhood level socioeconomic structure and the log of population density was specified as fixed effects while neighbourhood-level and parish-level unobserved geographical heterogeneity were specified as random effects. The models were constructed and evaluated using the statistical programming language R and the coxme packages [72,73].

## Results

**Fig 3:**
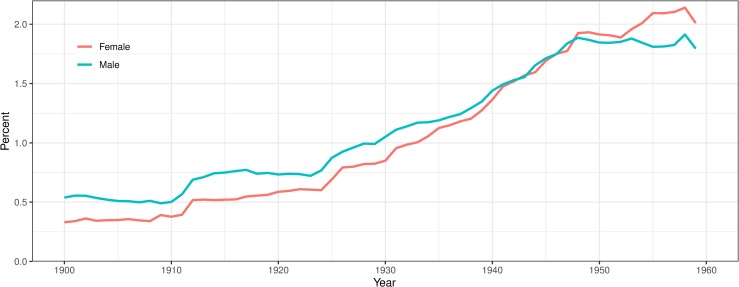
Gender-specific prevalence of mental disorders, in Västerbotten, Sweden, 1900–1959.

Fig 3 shows the prevalence rates of mental disorders by gender. Overall the prevalence of mental disorders increased over time, from around 0.4 percent to 1.9 percent of the population over 15. The general trend shows a modest increase until 1925 and then a stronger increase until the late 1940s, after which the prevalence stabilised. Men had a higher prevalence of mental disorders until the late 1950s, after which the proportion of women with mental disorders continued to increase. It should be noted that the changes in prevalences observed in the sample population corresponded with changes in confinement rates to mental hospitals among the total Swedish population over the same period which is what we expect given that a common reason for attaining a record of mental illness was being treated at a psychiatric institution [60,61].

**Fig 4:**
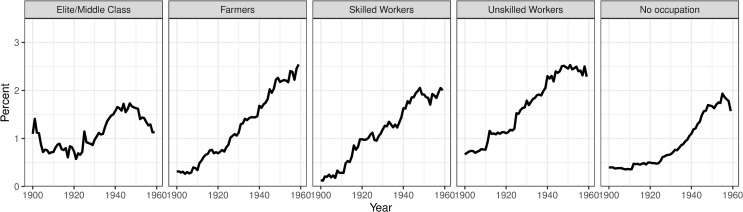
Mental disorder prevalence by SES, in Västerbotten, Sweden, 1900–1959.

In terms of socioeconomic differences, Fig 4 shows that the prevalence was relatively stable among the elite/middle-class (around 1 percent). During the study period prevalences increased for all other groups, most for farmers (from around 0.3 to 3 percent). In 1900, elite/middle-class had the highest rates while there were small differences among the other socioeconomic groups. In the 1950s, we observed larger variations in mental health by SES; the elite/middle-class had the lowest prevalence while farmers had the highest prevalence, followed by unskilled and skilled working classes.

**Fig 5:**
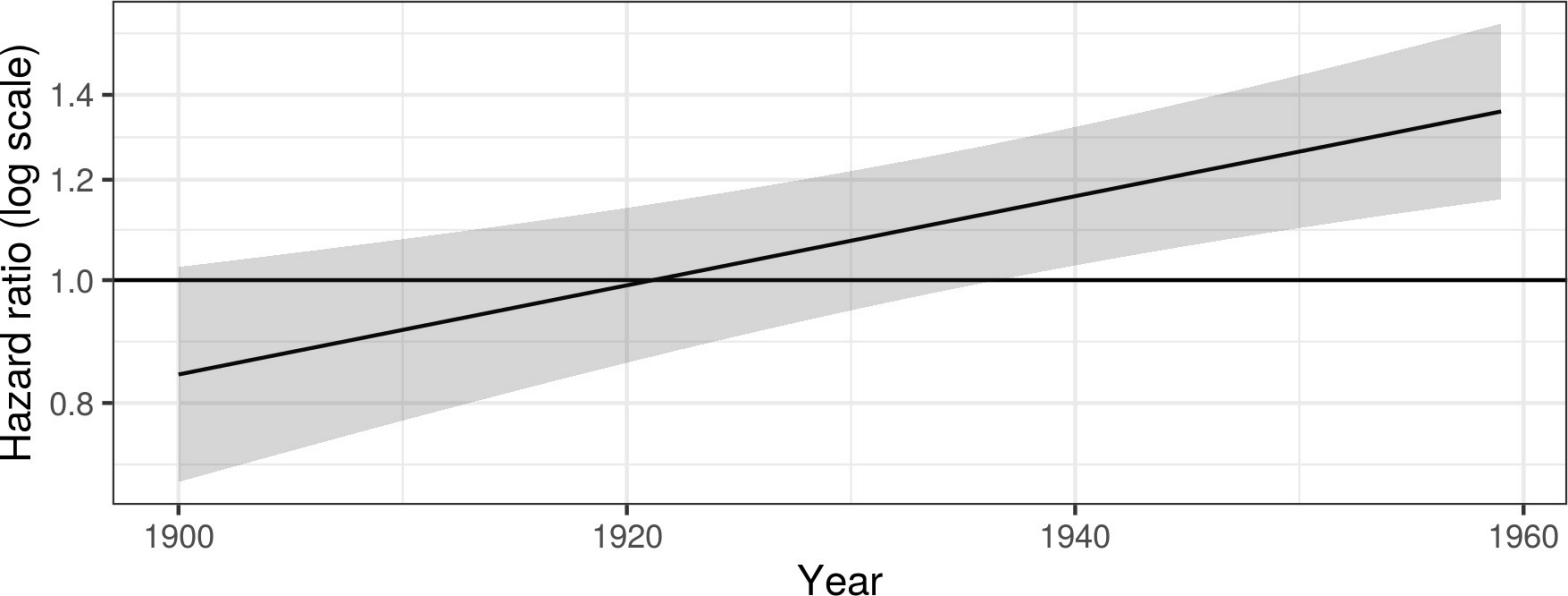
Predicted hazard ratio of mental disorder for women compared to men. Estimated using Cox proportional hazard models adjusted for individual and contextual factors. See S4 Table for further details on regression models.

Fig 5 shows the predicted relative hazard of mental disorder for women compared to men. After adjusting for compositional and contextual differences, women had approximately 14 percent lower risk of a mental disorder than men in 1900, but that gap between the genders closed over time and even resulted in a 37 percent higher risk for women in 1959 than men. Although the interactions showed significant differences for change over calendar time, the difference between genders at the beginning of the study period was not statistically significant.

**Fig 6:**
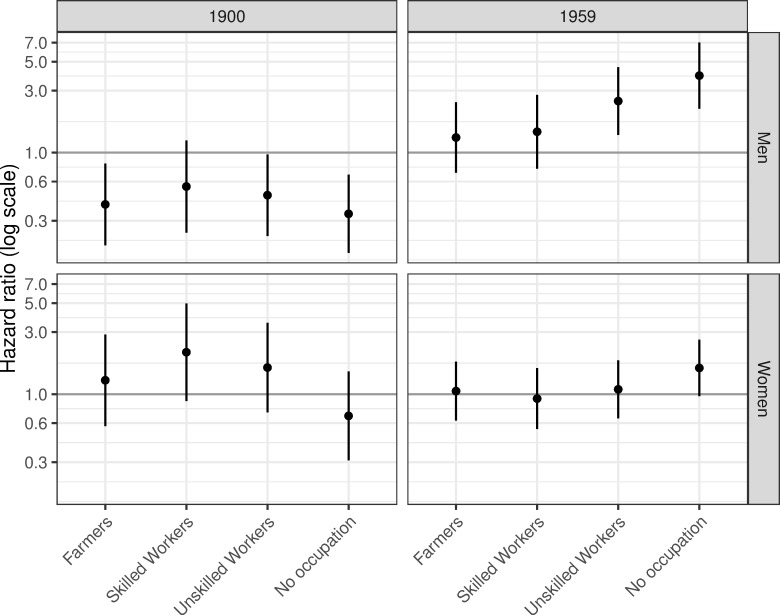
Predicted hazard ratio of mental disorder by SES compared to the elite/middle class, in 1900 and 1959 by gender. Estimates from gender-specific Cox proportional hazard models adjusting for individual and contextual level confounding factors. See S5 and S6 Tables for further details on regression models.

Among men, there was a reversal in the socioeconomic gradient in the risk of mental disorder (Fig 6). In 1900 elite/middle-class men had higher risks than all other groups, while men without an occupation had the lowest risks. All other SES groups had significantly lower risks, and the lowest SES groups had the lowest risks. Over time the relative differences reversed, and in 1959 elite/middle-class men had the lowest risks. At the end of the study period, we found significantly higher risks of mental disorder for male unskilled workers and those without an occupation. The results for women did not show the same pattern, as there were no significant differences between any SES groups over time. Although there was a continuous increase in risk over the period for women, there were no significant interactions between SES and calendar time, which indicated that there were no changes in hazard ratio between SES groups.

## Discussion

This study of Swedish longitudinal data demonstrates that contemporary socioeconomic and gender gaps in health emerged in the population between 1900 and 1959. In regards to gender gaps in mental disorders, we confirm the hypothesis that women ran higher risks than men, but only at the end of the study period. Similarly, we can confirm the hypothesis that low SES groups had a higher risk than high SES groups, but only at the end of the study period. Thus, we did not find that the associations were constant over time. Instead, we found that women had a lower risk of mental disorder than men in 1900, however, the differences are not statistically significant. Over time, the gap decreased, and in the 1950s the relative risk of women surpassed men. For men, we found a reversal of the socioeconomic gradient in the risk of mental disorder. In the early 1900s, the highest SES group experienced the highest risk while this had shifted to the opposite in the 1950s. Furthermore, we identified minimal differences in risks between the other socioeconomic groups in 1900, but these differences increased over time. For women, we identified much smaller SES differences, although low status increasingly became a risk factor. Our gender-specific results showed that SES had a greater impact on the probability of mental disorders among men than among women and that low social status increasingly became a risk factor for both genders.

Since the data stretches from 1900–1959 we could follow the historical changes in social inequalities in mental health further back in time than any previous study. However, we did not find the expected higher risks of mental disorders among women in 1900 as seen in most contemporary populations [1,2,5,6] or in studies of historical asylum populations [8,9]. The reversal in risks we identified in the early 20th century is instead consistent with historical studies on aggregate admission rates in England and Wales [10,11]. Additionally, this reversal suggests that any neurobiological sex differences contributing to women’s worse mental health [12,13] were significantly modified by social factors.

The inverse relationship between SES and mental health found in contemporary populations is related to both social causation and downward drift [1–7,20–22]. Due to the longitudinal design, our findings imply that the social causation mechanism did not contribute to the inverse relationship until the 1940s, in the sample population. Previous research on historical populations using treated samples show the expected inverse relationship [9,25–28]. These conflicting results suggest that downward social drift had a greater impact on SES inequalities in mental health in the 19th-century than social causation mechanisms. Furthermore, the positive association between mental disorders and SES in the early twentieth century uncovered among men in this study is mirrored in studies of long-term developments of SES differences in life expectancy. At least from the early 19th century until the mid 20th century, high-status men had lower survival chances than low-status men. As for women’s survival chances, there were small if any socioeconomic differences [33–35]. Our findings suggest that the SES reversal in survival in the 1970s was preceded by a reversal in the risk of mental disorder two decades earlier.

Furthermore, there seems to be a historically persistent age pattern in the age of onset of mental disorders. The reporting of mental disorders peaked around 25 in the historical sample, similarly to contemporary populations. This temporally fixed age pattern is consistent with neurobiological studies of mental disorders, which has found that the onset of mental disorders is related to the development of the brain, primarily during adolescence and young adulthood [64]. Differently from age, the effect of socioeconomic status and gender appear not to have been constant over time but rather, shifted in tandem with societal changes related to the development of Sweden into a modern welfare state. In this study we cannot determine what caused the emergence of modern gaps, merely report the changes in the individual level risk of mental disorders.

One potential explanation for the higher risk of mental disorders in high status groups in the early 20th-century is that the association between socioeconomic deprivation and high levels of psychosocial stress changed [24]. If that is the case, the results suggest that in early industrialised society, low SES was not associated with higher levels of stress that led to mental disorders, despite greater relative social deprivation. In 1900, Sweden was still a pre-welfare state and the household was the primary source for economic and social security. Social support was mainly provided through traditional networks of family and community [74]. Women had greater opportunities to fulfil expectations of their gender roles. Over the 60 years studied, the functioning of traditional social systems to cope with individuals’ distress diminished as the regions saw the establishment of large industries, a doubling of the population and greater urbanisation [38]. The rise in female workforce participation created a dual burden of responsibilities for both work and the household, increasing exposure to stress in particular among women [15,75,76]. The emerging welfare state, from the 1930s, might have been unable to provide social and emotional support for individuals in the lower social strata, to the same extent that family and social networks were able to do in the more traditional agricultural society.

Another potential explanation is that the emergence, or potential shift in mental health gaps, reflect a change in how mental disorders were expressed and perceived at the time. Through an increased medicalisation of mental health, the line between normal and deviant was moved [77,78]. That men had higher risks in the early 20th century is consistent with historical studies of madness and gender. Historians argue that men were perceived as the mentally weaker sex and madness was associated with masculinity. The reversal has been argued to occur during the 19th century as female mental health was increasingly pathologized [79–81]. Furthermore, the medicalisation of mental disorders is not equally distributed over socioeconomic groups. There are large SES variations in the prevalence of different mental disorders and social inequalities in the treatment of mental disorders is well documented [30]. The changes in social inequalities in mental disorders reported in this study were accompanied not only by an overall increase in mental disorder prevalence, but also by an expansion of the Swedish mental health care system. In Sweden, the number of beds in mental hospitals expanded from around 4,600 beds in 1900 to around 16,000 in the 1960s, and the confinement rates among women increased more rapidly compared to men [60,61]. The results could thus be linked to an increased pathologization of the mental health of women and low SES groups. However, the two explanations are not necessarily contradictory. The expansion of institutional mental health care in the late 19th and early 20th century has also been explained by a greater need for institutional care as traditional systems of social support eroded [27].

The findings in this study must be considered in light of several limitations. First, it must be borne in mind that our identification of mental disorders depended upon impairment notes made by church officials who were not trained as medical professionals. However, the age-distribution of first reported mental disorders in our historical sample is similar to contemporary populations suggesting that the reporting reflect similar types of distress as in modern data. As already mentioned the most common reason for reporting a mental illness in the population register was that the individual had been treated at a psychiatric institution. That makes the reporting strongly dependent on the diagnostic work done by medical doctors. Additionally, the tendency for reporting higher rates among men and higher status individuals in the early period is contrary to what we would expect if the reporting was socially biased. Church officials might have had more reasons to be hesitant of reporting socially stigmatised conditions such as mental disorders regarding individuals that had higher social status and moved in the same social circles as themselves. In the local community, the minister and the other civil servants that were responsible for keeping church records held a relatively high social status at the time. The fact that we found the opposite pattern of higher rates of mental disorders in the upper social strata makes it highly unlikely that such social concerns influenced this type of health documentation. Rather it suggests that the legal obligation that the church officials had to keep correct and up to date records of mental illness in the population was something that the minister did to the best of his abilities and knowledge.

Second, we relied on reported mental disorders and not experienced distress. Reported disorders do not always reflect actual experiences, and men's mental distress is often underestimated in official reporting [82]. In contemporary populations, there are significant class and gender-specific diagnosis biases of mental disorders and we have no reason to assume that this was not equally the case during the period we study here [83]. Furthermore, we cannot determine whether higher mental disorder risks in high status groups in the early twentieth century was caused by higher levels of distress than in lower status groups. It might very well be that during this early period high-status individuals experienced a lower bar for what was regarded as unacceptable expressions of emotions, thinking and behaviours that resulted in a higher probability to be registered with a mental disorder. Another factor that might have contributed to this is that high-status families were potentially more prone to seek help for family members from mental care facilities than working-class families.

Third, the results of this study are limited to SES derived from occupational titles. Other SES indicators have been shown to have different gendered impacts, such as wealth or income [6]. Moreover, the study was based on a geographically limited population. There might be differences between locations caused by contextual factors. Finally, despite the adjustment of measures of both individual and contextual differences, there remains the possibility for unmeasured confounders, especially regarding individual and collective social support.

## Conclusions

Overall, our longitudinal study of a Swedish population, observed 1900–1959, suggests that the socioeconomic and gender gaps in mental disorders that exist today are not historically persistent, but emerged during the period of rapid modernisation of Swedish society in 1940–1959. Consistent with previous studies on contemporary populations, women ran higher risks than men, and low SES groups had higher risks than high SES groups in the 1950s, however this was not the case at the beginning of the century. In 1900, women had a lower risk than men and, for men, the relationship between SES and mental disorders was positive. Our longitudinal study shows empirical evidence that is exceptional in suggesting that, under certain circumstances, female gender or low SES must not imply higher risks of being perceived as mentally ill.

These findings need to be considered by future research. Studies of social inequalities in mental health cannot assume that these inequalities have always existed. This knowledge should be used to try to understand why the reverse gradient existed in historical populations and what factors contributed to this pattern, whether it was an effect of increased medicalisation or increased exposure to stress factors among women and low SES groups. Furthermore, little is known about the potential role of downward drift in creating observed historical inequalities. Historical studies of mental health can show how social and cultural contexts influence the association between mental health, gender and socioeconomic status. Such insights are essential for understanding why inequalities in mental health emerge, persist and possibly how such inequalities could be limited.

## Supporting information

S1 Table Socioeconomic classification scheme. (PDF)

S2 Table Descriptive statistics of total study population and thouse ever having a mental disorder in 12 parishes, Västerbotten county, 1900–1959. Subjects enter from age 15 onward and followed until attaining a mental disorder or they are censored. Values calculated at the reporting of a mental disorder or at mean follow up time.
(PDF)

S3 Table Descriptive statistics of geographical units. (PDF)

S4 Table Hazard Ratio (HR) of mental disorder in Västerbotten 1900–1959. Interaction between gender and calendar time.
(PDF)

S5 Table Hazard Ratio (HR) of mental disorder for men in Västerbotten 1900–1959. (PDF)

S6 Table Hazard Ratio (HR) of mental disorder for women in Västerbotten regions 1900–1959. (PDF)
