# Supplementary material for: The emergence of social gaps in mental health: A longitudinal population study in Sweden, 1900-1959
Source: PLoS One. 2020 Apr 30;15(4):e0232462. doi: 10.1371/journal.pone.0232462 (PMC7192474; doi:10.1371/journal.pone.0232462)
Supplement: S1 Table — (PDF) [file pone.0232462.s001.pdf]

S1 Table: Socioeconomic classification scheme.

Nr	SOCPO	Condensed SOCPO applied in the study
6	Elite	Elite/Middle Class
5	Middle Class	
4	Farmers	Farmers
3	Skilled Workers	Skilled Workers
2	Semi-skilled Workers	
1	Unskilled Workers	Unskilled Workers
0		No occupation
