# Supplementary material for: The emergence of social gaps in mental health: A longitudinal population study in Sweden, 1900-1959
Source: PLoS One. 2020 Apr 30;15(4):e0232462. doi: 10.1371/journal.pone.0232462 (PMC7192474; doi:10.1371/journal.pone.0232462)
Supplement: S2 Table — Subjects enter from age 15 onward and followed until attaining a mental disorder or they are censored. Values calculated at the reporting of a mental disorder or at mean follow up time. (PDF) [file pone.0232462.s002.pdf]

S2 Table: Descriptive statistics of total study population and thouse ever having a mental disorder in 12 parishes, Västerbotten county, 1900-1959. Subjects enter from age 15 onward and followed until attaining a mental disorder or they are censored. Values calculated at the reporting of a mental disorder or at mean follow up time.

		**Total population**		**With a mental disorder**	
**Variable**	**Level**	**Mean/Percent**	**Count**	**Mean/Percent**	**Count**
Mental disorder		0.01		1.00	
Individuals			193893		2450
Age at entry		20.31		19.08	
Age at exit		40.43		38.90	
Follow-up time		20.12		19.82	
Gender	Man	48.00	93766	48.00	1170
	Women	52.00	100127	52.00	1280
Marital status	Married	49.00	94752	38.00	942
	Divorced	0.00	741	0.00	10
	Unmarried	46.00	89314	59.00	1441
	Widowed	5.00	9086	2.00	57
Migrant	False	38.00	73810	47.00	1151
	True	62.00	120083	53.00	1299
SES	Elite/Middle Class	9.00	17710	5.00	122
	Farmers	22.00	42899	20.00	502
	Skilled Workers	18.00	34460	12.00	296
	Unskilled Workers	23.00	45172	23.00	562
	No occupation	28.00	53652	40.00	968
Local SES structure	Urban	11.00	21488	13.00	321
	Semi-urban	54.00	103777	34.00	835
	Working-class	4.00	8401	9.00	213
	Rural	25.00	47582	35.00	860
	None	7.00	12645	9.00	221
Log of population density		3.47	193893	3.67	2450
Calendar time	1900-1919	17.00	32155	16.00	397
	1920-1939	22.00	41855	39.00	960
	1940-1959	62.00	119883	45.00	1093
