# Supplementary material for: The emergence of social gaps in mental health: A longitudinal population study in Sweden, 1900-1959
Source: PLoS One. 2020 Apr 30;15(4):e0232462. doi: 10.1371/journal.pone.0232462 (PMC7192474; doi:10.1371/journal.pone.0232462)
Supplement: S3 Table — (PDF) [file pone.0232462.s003.pdf]

S3 Table: Descriptive statistics of geographical units.

**Unit**	**Variable**	**Mean**	**25th**	**Median**	**75th**
Parish (N=12)	Population	9331.16	5083.5	7082	12853.5
	Area (Square kilometers)	1106.41	332.13	1131.42	1755.75
	Density (Population/Sq.km.)	17.19	3.35	12.07	17.7
Neighbourhood (N=229)	Population	350.98	58	127	298
	Area (Square kilometers)	42.98	16.45	27.81	54.74
	Density (Population/Sq.km.)	11.58	1.73	3.51	9.78
