# Supplementary material for: The emergence of social gaps in mental health: A longitudinal population study in Sweden, 1900-1959
Source: PLoS One. 2020 Apr 30;15(4):e0232462. doi: 10.1371/journal.pone.0232462 (PMC7192474; doi:10.1371/journal.pone.0232462)
Supplement: S4 Table — Interaction between gender and calendar time. (PDF) [file pone.0232462.s004.pdf]

S4 Table: Hazard ratio (HR) of mental disorder in Västerbotten 1900-1959. Interaction between gender and calendar time.

	Model 1		Model 2		Model 3	
**Variable**	**HR**	**P-value**	**HR**	**P-value**	**HR**	**P-value**
Women	0.908	0.350	0.888	0.259	0.876	0.210
Calendar time, centered at 1900	1.016	<0.001	1.021	<0.001	1.023	<0.001
Women * Calendar time	1.006	0.035	1.007	0.009	1.008	0.006
Divorced			2.485	0.004	2.428	0.005
Unmarried			1.939	<0.001	1.932	<0.001
Widowed			1.026	0.856	1.046	0.753
Farmers			0.986	0.890	1.002	0.987
No occupation			1.256	0.022	1.252	0.025
Skilled Workers			1.114	0.315	1.108	0.340
Unskilled Workers			1.266	0.020	1.303	0.009
Migrant			0.849	<0.001	0.798	<0.001
Local SES: Urban			0.942	0.494	1.024	0.913
Local SES: Semi-urban			0.696	<0.001	0.558	0.008
Local SES: Working-class			1.349	0.003	1.428	0.110
Local SES: Rural			1.069	0.393	1.115	0.609
Log of population density			0.941	<0.001	0.896	<0.001
SD Neighborhood-level effect					0.198	
SD Parish-level effect					0.231	
N Neighborhoods					229.000	
N Parishes					12.000	
N individuals	193893		193893		193893	
Events	2450.000		2450.000		2450.000	
Log likelihood	-27055.176		-26889.222		-26796.035	
P-value	<0.001		<0.001		<0.001	
AIC	54116.352		53810.444		53714.932	
