# Supplementary material for: The emergence of social gaps in mental health: A longitudinal population study in Sweden, 1900-1959
Source: PLoS One. 2020 Apr 30;15(4):e0232462. doi: 10.1371/journal.pone.0232462 (PMC7192474; doi:10.1371/journal.pone.0232462)
Supplement: S5 Table — (PDF) [file pone.0232462.s005.pdf]

S5 Table: Hazard ratio (HR) of mental disorder for men in Västerbotten 1900-1959.

	Model 1		Model 2		Model 3	
**Variable**	**HR**	**P-value**	**HR**	**P-value**	**HR**	**P-value**
Farmers	0.445	0.023	0.378	0.008	0.400	0.013
No occupation	0.398	0.007	0.328	0.001	0.339	0.002
Skilled Workers	0.552	0.143	0.551	0.148	0.550	0.150
Unskilled Workers	0.575	0.122	0.453	0.030	0.471	0.040
Calendar time, centered at 1900	0.987	0.146	0.992	0.357	0.993	0.435
Farmers * Calendar time	1.023	0.020	1.022	0.033	1.020	0.052
No occupation * Calendar time	1.043	<0.001	1.043	<0.001	1.042	<0.001
Skilled Workers * Calendar time	1.018	0.110	1.017	0.135	1.017	0.147
Unskilled Workers * Calendar time	1.029	0.004	1.029	0.004	1.029	0.005
Migrant			0.744	<0.001	0.707	<0.001
Divorced			4.492	<0.001	4.260	<0.001
Unmarried			2.101	<0.001	2.109	<0.001
Widowed			1.038	0.872	1.046	0.846
Local SES: Urban			0.953	0.700	1.037	0.887
Local SES: Semi-urban			0.693	0.001	0.606	0.050
Local SES: Working-class			1.461	0.008	1.515	0.116
Local SES: Rural			1.103	0.377	1.135	0.607
Log of population density			0.925	0.002	0.890	<0.001
SD Neighborhood-level effect					0.219	
SD Parish-level effect					0.165	
N Neighborhoods					229.000	
N Parishes					12.000	
N individuals	93766		93766		93766	
Events	1170.000		1170.000		1170.000	
Log likelihood	-12115.064		-12023.975		-11973.224	
P-value	<0.001		<0.001		<0.001	
AIC	24248.128		24083.951		24047.628	
