# Supplementary material for: The emergence of social gaps in mental health: A longitudinal population study in Sweden, 1900-1959
Source: PLoS One. 2020 Apr 30;15(4):e0232462. doi: 10.1371/journal.pone.0232462 (PMC7192474; doi:10.1371/journal.pone.0232462)
Supplement: S6 Table — (PDF) [file pone.0232462.s006.pdf]

S6 Table: Hazard ratio (HR) of mental disorder for women in Västerbotten regions 1900-1959.

	Model 1		Model 2		Model 3	
**Variable**	**HR**	**P-value**	**HR**	**P-value**	**HR**	**P-value**
Farmers	1.036	0.929	1.172	0.697	1.279	0.551
No occupation	0.732	0.424	0.665	0.303	0.681	0.338
Skilled Workers	1.826	0.158	2.116	0.085	2.099	0.092
Unskilled Workers	1.580	0.245	1.501	0.310	1.601	0.244
Calendar time, centered at 1900	1.019	0.039	1.025	0.007	1.027	0.004
Farmers * Calendar time	1.001	0.921	0.998	0.873	0.997	0.755
No occupation * Calendar time	1.017	0.083	1.015	0.125	1.014	0.154
Skilled Workers * Calendar time	0.989	0.301	0.986	0.196	0.986	0.207
Unskilled Workers * Calendar time	0.994	0.519	0.994	0.564	0.993	0.523
Migrant			0.936	0.289	0.861	0.024
Divorced			1.582	0.362	1.565	0.373
Unmarried			1.867	<0.001	1.859	<0.001
Widowed			1.024	0.894	1.051	0.782
Local SES: Urban			0.942	0.628	1.004	0.984
Local SES: Semi-urban			0.723	0.003	0.596	0.003
Local SES: Working-class			1.253	0.117	1.262	0.231
Local SES: Rural			1.051	0.651	1.080	0.647
Log of population density			0.955	0.052	0.922	0.010
SD Neighborhood-level effect					0.125	
SD Parish-level effect					0.209	
N Neighborhoods					229.000	
N Parishes					12.000	
N individuals	100127		100127		100127	
Events	1280.000		1280.000		1280.000	
Log likelihood	-13178.598		-13121.299		-13082.936	
P-value	<0.001		<0.001		<0.001	
AIC	26375.197		26278.598		26236.882	
